# Revisiting the History of Candidiasis

**DOI:** 10.7759/cureus.78878

**Published:** 2025-02-11

**Authors:** Savita B Tajane, Satyajeet Pawar, Satish Patil

**Affiliations:** 1 Department of Microbiology, Krishna Institute of Medical Sciences, Krishna Vishwa Vidyapeeth, Karad, IND

**Keywords:** antifungal resistance, candida, candidiasis, historical perspectives, virulence

## Abstract

Candida infections in humans are well-documented, but their clinical manifestations have become more evident in recent years. This shift is attributed to advancements in diagnostic modalities and the increasing use of aggressive therapies, where patients are exposed to more invasive procedures, broad-spectrum antibiotics, and immunosuppressive drugs. This shift is attributed to advancements in diagnostic modalities and the increasing use of aggressive therapies, where patients are exposed to more invasive procedures, broad-spectrum antibiotics, and immunosuppressive drugs. This review article outlines various historical milestones regarding multiple aspects of Candida, including its taxonomy, epidemiology, pathogenicity, and therapeutic and diagnostic modalities. A thorough search of the available literature was conducted using scientific search engines such as Google Scholar, PubMed, and ScienceDirect, utilizing appropriate Medical Subject Headings terms. Relevant research articles highlighting important historical milestones related to Candida's taxonomy, pathogenicity, clinical manifestations, diagnostic modalities, and treatment were retrieved and utilized to prepare this manuscript. The historical perspective of Candida is dynamic, and while many aspects have become clear over the years, there are still numerous areas that need further elucidation to fully understand the complex mechanisms of pathogenicity, epidemiology, and antifungal resistance. Additionally, this review aims to provide new insights that will support the development of novel antifungal molecules to expand the current antifungal armamentarium against Candida, which, at present, is limited to three classes: azoles, polyenes, and echinocandins.

## Introduction and background

A notable increase in national and international research publications in recent years provides direct evidence that candidiasis is gaining significance. Candida's involvement in human infections is not a new concept. Certain infections, particularly those linked to the proliferation of Candida spp., have been documented for a long time. However, in the last few decades, the incidence of disseminated infections, once rarely reported, has significantly increased.

Certain clinical manifestations, such as meningitis, septic arthritis, septicemia, and osteomyelitis, where the involvement of Candida spp. was once seldom observed, have become more evident in recent years. This can be attributed to advancements in medicine, where patients are subjected to more aggressive treatments, and developments in diagnostic mycology, which have increased the determination of fungal pathogens [1].

In recent years, the overall epidemiology of Candida infections has experienced a significant shift, with many species, which were previously considered nonpathogenic and saprotrophic, emerging as important causes of more serious and treatment-resistant infections. Empirical antifungal therapy, in general, and the rampant, injudicious use of fluconazole in particular, along with an increase in the number of immunocompromised patients, are cited as important risk factors contributing to the changes in the epidemiology of candidiasis [2]. This review article outlines various historical milestones pertaining to multiple aspects of Candida, including its taxonomy, epidemiology, pathogenicity, and therapeutic and diagnostic modalities. This will help to understand how various aspects of Candida infections have changed over time and will additionally provide better future insights for understanding the factors responsible for the emergence of drug resistance in Candida and the alteration in the epidemiology of candidiasis.

## Review

Search strategy

To prepare this review article, which explores the historical perspective of various medical aspects of Candida, a comprehensive literature search was conducted using search engines such as Google Scholar, PubMed, and ScienceDirect. The search utilized Medical Subject Headings terms, including “history of Candida”, “taxonomy of Candida”, “Candidiasis”, “virulence/pathogenicity of Candida”, “laboratory diagnosis of Candidiasis”, and “treatment of Candidiasis”. Relevant reference articles in the English language were retrieved from these search engines. The information gathered from these research manuscripts was then compiled and used to prepare this article.

History of Candida

Oral candidiasis, caused by Candida spp., was first documented by Hippocrates in 377 BCE. He referred to it as "aphthae" or thrush [3], a term believed to derive from an ancient Scandinavian or Anglo-Saxon word. Hippocrates recorded his observations on oral candidiasis in his work Epidemics, which was published in the fourth century BC [3].

Oral Candida infections were also documented by Galen in the second century AD when he referred to the condition as "aphthas albus." Galen observed oral candidiasis (thrush) in sick children. Additionally, this disease was recorded in Pepys' diary in 1665 [3]. During this period, however, there was considerable uncertainty regarding the etiology of candidiasis. There was ambiguity about whether the infection was primarily host-related, caused by an infectious agent, or a combination of both.

Researchers such as Rosen von Rosenstein (1771) and Underwood (1784) supported the idea that an infectious agent is involved in oral thrush [3]. Rosen von Rosenstein also described an invasive form of thrush, although the etiological agent was not specified [3]. In 1786, the high prevalence of oral thrush led the French Société Royale de Médecine to offer a prize of 1,299 pounds for research into oral candidiasis [4,5].

In 1835, Vernon reported the first case of esophageal candidiasis in a neonate, suggesting that the infection was acquired during passage through the birth canal. In 1839, Langenbeck identified a fungal agent in a typhoid patient with oral thrush, referring to it as a "Cryptogamic Plant." However, he attributed it to causing fever rather than thrush [5]. The word “Cryptogam” is derived from the Greek words “Kryptos,” meaning hidden, and “Gam,” meaning marriage. It was named due to the appearance of Candida as "long branched tubes and round or oval globules," resembling parts of plants like branches and seeds. Three years later, in 1842, David Gruby identified the fungal agent of thrush, naming it le vrai Muguet des enfants, and initially classified it under the genus Sporotrichum. Later on, it was reclassified as the genus contains only filamentous fungi. The placement of Candida in the genus Sporotrichum may be due to polymorphism, where this organism demonstrates a variety of morphology, such as yeast, budding yeast, and pseudohyphae. The pseudohyphal stage of the Candida may have been responsible for misplacement in the genus Sporotrichum [3,4]. Similarly, the genus Sporotrichum contains saprophytic fungi without any pathogenic role. Gruby was the first to provide a written document describing cells of thrush fungus and comparing it with those of fungus causing dermatophytosis.

In 1844, Bennett identified the same fungus in the sputum and lungs of a tuberculosis patient with pneumothorax [5]. He was also the first to explore the connection between *Candida albicans* and thrush by inoculating healthy infants with material from aphthous membranes. Bennett documented the microscopic features of the fungus, which appeared to be *C. albicans*. Like Robin, he also suggested that debilitation could be a predisposing factor for candidiasis [5].

In 1846, Berg definitively established that a fungus was the causative agent of thrush and further confirmed his hypothesis by successfully reproducing the infection in healthy children, thereby validating that thrush is indeed a fungal infection [5]. In 1849, Wilkinson documented cases of vaginal thrush [3], and a year later, Virchow described the involvement of thrush fungi in subcutaneous infections. In 1851, Bonorden isolated a fungus from decaying wood and named it *Monilia albicans* [3].

In 1853, Charles Philippe Robin observed that the "thrush" fungus could lead to systemic infections in terminally ill patients [5,6]. He introduced the binomial name *Oidium albicans* for the thrush fungus, with the generic name Oidium reflecting the yeast cell’s egg-like or oval shape. Robin was the first to use the term albicans, which refers to "whitening" [3]. He also described both the yeast and filamentous forms of the fungus and proposed that debilitation could be a predisposing factor for candidiasis [3,4].

In 1862, Zenker was the first to report a case of systemic candidiasis [3]. In 1868, Hansen described the thrush fungus as dimorphic, exhibiting two distinct morphological forms: yeast and mycelia. However, Hansen continued to use *Monilia candida* as the valid name for the fungus [3,6]. In 1875, Haussman demonstrated that the causative agent of both oral and vaginal thrush was the same and showed that the fungus could be transmitted from a mother's vaginal lesion to the infant's mouth [3,4]. Grawitz (1874) also affirmed Hansen's observations by describing the dimorphic characteristics of the thrush fungus [3].

In 1887, Plaut induced experimental lesions in the throats of chickens by inoculating them with a fungus isolated from decaying wood, confirming it as the etiological agent of oral thrush. In the same year, Audrey supported the concept of dimorphism, proposing that the two distinct morphological forms of the same fungal species resulted from environmental conditions [3].

In 1890, Zopf renamed the thrush fungus *M. albicans* and referred to the infection as moniliasis. The term "candidiasis," which is now used for Candida infections, was derived from "moniliasis" [3]. In 1912, Castellani described several new species of Candida, including *Candida guilliermondii*, *Candida kefyr*, and *Candida tropicalis* [3]. Despite these discoveries, Castellani strongly advocated for retaining the name Monilia, and under his influence, this term became widely used in medical literature [3].

In 1923, Berkhout resolved the confusion regarding the nomenclature of thrush fungi by introducing the generic name Candida. The name Candida originates from the Latin phrase toga candida, meaning "white toga," referring to the white robe worn by candidates for the Roman Senate [3,6]. Berkhout chose the term Candida due to the white appearance of its colonies. This term was used to describe small, colorless yeasts with minimal hyphal formation, often appearing flat and breaking into shorter and longer segments. Yeasts in the Candida genus produce conidia through budding, either on hyphae or from other yeast cells [5,6]. However, many researchers like Langeron and Talice doubted the term “Candida” proposed by Berkhout and suggested including Candida spp. into eight different genera like “Blastodendrion,” “Candida,” “Geotrichoides,” “Geotrichum,” “Monilia,” “Mycocandida,” “Mycotorula,” and “Mycotoruloides” [6].

In 1940, Martin and Jones contributed to nomenclatural confusion by misclassifying Candida under the genus Monilia, a group of fungi commonly found on plants. As a result, many clinicians mistakenly referred to thrush as "moniliasis," despite the clear morphological differences between the thrush fungus and Monilia, which had already been clarified by mycologists [6]. The generic name Candida was officially accepted as a nomen conservandum by the Eighth Botanical Congress in Paris in 1954. “Nomen conservandum” is a Latin term that means “name to be conserved” and is interchangeably used by the “International Code of Nomenclature for Algae, Fungi, and Plants.” The specific epithet albicans is derived from the Latin word albicare, meaning "to whiten" [6]. Initially, *C. albicans* was thought to cause only oral and vaginal thrush. However, it was later found to be associated with a wide range of clinical conditions, including bloodstream infections, central nervous system infections, onychomycosis, and dermatitis [7,8]. In 1933, Smith and Sano documented the first case of meningitis caused by Candida [9]. Subsequent studies led to isolating several non-albicans Candida (NAC) species from human infections. Anderson (1917) reported *Candida glabrata*, while Ashford (1928) identified *Candida parapsilosis* in a case of diarrhea [10,11]. In Indian hospitals, *C. parapsilosis *is the second most common cause of disseminated infection, including candidemia. It is particularly common in critically ill patients, neonates, and patients with invasive medical devices. This pathogen has a unique property of multiplying in hyperalimentation solutions. In 1979, Pappagianis et al. documented the first instance of human candidiasis caused by *Candida lusitaniae* [12].

Lavarde et al. (1984) isolated *Candida haemulonii* from the blood culture of a patient who succumbed to kidney failure [13]. In 1995, Sullivan and Coleman identified a new species, *Candida dubliniensis*, from cases of oral candidiasis in human immunodeficiency virus-infected patients in Dublin, Ireland [14]. In the same year, Rex et al. (1995) reported fungemia caused by *Candida lipolytica* in a cancer patient undergoing parenteral fluconazole treatment [15].

Overview of initial isolation reports of NAC species (emerging pathogens)

The details of different NAC species, for the first time described from various clinical manifestations, are shown in Table 1.

**Table 1:** Non-albicans Candida spp. described for first time in different clinical manifestations

Non-albicans Candida spp.	Researcher and year of discovery	Clinical manifestation
Candida guilliermondii	Castellani (1912) [3]	Bloodstream infection
Candida kefyr	Castellani (1914) [4]	Infection of the ear, gastrointestinal tract, and vagina
Candida glabrata	Anderson (1917) [10]	Bloodstream infection
Candida parapsilosis	Ashford (1928) [11]	Diarrhea
Candida lusitaniae	Pappagianis et al. (1979) [12]	Bloodstream infection
Candida haemulonii	Lavarde et al. (1984) [13]	Bloodstream infection in a renal failure patient
Candida dubliniensis	Sullivan et al. (1995) [14]	Oral candidiasis
Candida lipolytica	Rex et al. (1995) [15]	Bloodstream infection
Candida tropicalis	Castellani (1910) [16]	Bloodstream infection
Candida krusei	Castellani (1914) [17]	Bloodstream infection
Candida viswanathii	Randhawa and Chowdhary (1959) [18]	Fatal case of meningitis
Candida ciferrii	Gunsilius et al. (2001) [19]	Bloodstream infection
Candida auris	Satoh et al. (2009) [20]	Infected ear canal

Development of mycology and candidiasis: the Indian Scenario

In 1957, the Vallabhbhai Patel Chest Institute (VPCI) in New Delhi set up a dedicated mycology diagnostic unit. Under the leadership of Professor Raman Viswanathan, the Founder-Director of the institute, the unit worked on an Indian Council of Medical Research-funded project focused on bagassosis [18].

In 1959, Viswanathan and Randhawa from VPCI identified a novel yeast species in the cerebrospinal fluid of a patient who succumbed to meningitis. A similar yeast species was later isolated by Sandhu and Randhawa in 1960 from the sputum of another patient [18,21]. This yeast was eventually classified as a new species of Candida and named *Candida viswanathii *in recognition of the visionary leadership of Professor Viswanathan, the Founder-Director of the institute [18,21]. In 1965, Sandhu et al. demonstrated the pathogenic potential of *C. viswanathii* through laboratory animal studies, suggesting its role as a potential pathogen [18,21]. However, this NAC spp. is rarely encountered in human infections, and to recent date, only 17 cases have been reported worldwide. Although *C. viswanathii* is isolated from various environmental sources, its isolation is not reported from the hospital environment and hands of healthcare workers.

In 2003, Chowdhary et al. from Kalawati Saran Children’s Hospital, New Delhi, were the first to report a nosocomial outbreak of candidemia caused by *C. tropicalis* in neonates, demonstrating the clonal origin of the isolates. This study underscored the growing prevalence of NAC spp. in neonatal candidemia cases [22].

Two significant events in the history of medicine have greatly influenced the overall epidemiology of mycotic infections, particularly candidiasis. The first was the introduction and widespread use of broad-spectrum antibiotics, which promoted Candida infections by suppressing bacterial commensals. The second was the notable rise in the number of immunocompromised patients, which increased the prevalence of Candida infections and led to the emergence of less virulent yet more treatment-resistant NAC spp. [23]. In 1980, Whelan et al. established the foundation for molecular techniques in Candida by developing a parasexual genetic system utilizing a cloned *C. albicans* ADE2 gene [24]. Subsequently, in 1986, Kurtz et al. introduced a DNA-mediated transformation system [25].

Risk factors for Candidiasis

The most common risk factors for candidiasis are long-term usage of broad-spectrum antimicrobial agents, compromised immune status (both induced and acquired), surgical intervention, central venous catheters, total parenteral nutrition, solid organ and bone marrow transplant, chemotherapy, diabetes, prolonged hospital and ICU stay, and extremes of the age of life [2].

Discovery of antifungal drugs and methods for AFST

In 1953, Gold et al. discovered amphotericin B, the first broad-spectrum antifungal agent in the polyene class [26]. In the 1990s, ketoconazole, an oral imidazole, was introduced, followed by the licensing of fluconazole for the prophylaxis and treatment of various types of candidiasis [27]. Nystatin, a topical polyene antifungal, was introduced in 1992. Echinocandins are the most recent addition to the antifungal armamentarium against Candida spp. To date, three echinocandins are approved by the Food and Drug Administration: caspofungin, micafungin, and anidulafungin. Echinocandins, especially caspofungin, are used as the first line of treatment in invasive Candida infections. This class of antifungal agents has also been reported to be effective in treating infection due to *Candida auris*, a treatment-resistant species. However, echinocandin resistance is reported in a few strains of* C. auris*. In developing countries like India, widespread use of echinocandins is usually restricted due to their high cost.

The emergence of NAC species underscored the necessity for antifungal susceptibility testing (AFST). In 1982, the Clinical and Laboratory Standards Institute (CLSI) introduced the broth macrodilution technique, which was officially approved in 1997 for evaluating antifungal susceptibility in yeasts and yeast-like fungi [28]. This method was later superseded by the microdilution broth method, which offered a more streamlined and efficient approach. The CLSI microdilution broth method (BMD) is regarded as the standard reference for AFST [28]. The agar disc diffusion method for assessing Candida susceptibility to antifungal agents was first proposed by Meis et al. (2002) and subsequently modified by Barry et al. (2002) [29]. In addition to the CLSI reference BMD method (M27), the disc diffusion method (M44) is approved for AFST of Candida spp. It provides both qualitative interpretive criteria (susceptible, susceptible dose-dependent, intermediate, resistant) and quantitative measurements (zone of inhibition) [28]. Later on, various commercial techniques (automatic and semiautomatic) were developed for antifungal testing of Candida spp.

*C. auris*: a novel addition to pathogenic Candida spp.

In 2009, Satoh et al. identified a novel ascomycetous yeast from the external ear canal of a patient at a Japanese hospital. This yeast was subsequently named *C. auris* based on its initial isolation from the ear canal [20]. Although *C. auris* was first isolated from the ear canal, it has now been identified in healthcare-related bloodstream infections. This species, which has been recognized for over a decade, is known for its resistance to treatment and is linked to increased mortality rates. In India, this pathogen has recently been reported in various healthcare setups. As this pathogen can easily spread in a hospital environment, strict adherence to infection prevention and control practices is essential. It is also evident that *C. auris* reported from outbreak clonal differ genotypically and phenotypically from Korean and Japanese isolates.

Virulence factors: the attributors to the pathogenicity of Candida spp.

Candida is a highly versatile organism, capable of existing in both harmless and pathogenic states in the human body. It thrives in various environmental conditions, such as on hospital floors, surfaces, parenteral nutrition solutions, and medical equipment. Furthermore, it exhibits the ability to transition between different morphological forms [30]. In the past, Candida was believed to play a passive role in the development and progression of infections, with the underlying cause attributed to organic weakness. However, this view has evolved, and it is now well-established that Candida spp. actively contribute to the onset and advancement of infections through various mechanisms known as "virulence factors" [2].

Adhesion, biofilm formation, extracellular enzymes, and hemolytic properties are among the most studied virulence traits of Candida spp. In 1994, Hostetter identified the adhesive properties of Candida and proposed three types of adhesive interactions: protein-protein interaction, lectin-like interaction, and incompletely defined interactions [31]. Staib was the first to report proteinase activity in Candida spp. In 1980, Odds and Abbott suggested a simple technique for detecting extracellular proteinase activity in Candida spp. using bovine serum albumin agar [32]. Costa et al. and Werner were the first to report secretory phospholipase enzymes in *C. albicans*, whereas Price et al., in 1982, developed an egg yolk agar-based in vitro technique for detecting phospholipase [33,34].

In 1965, Werner reported extracellular lipase activity in *C. albicans*, whereas the first gene responsible for lipase activity (L1P1) was detected by Fu et al. in 1997 [35]. In 2001, Luo et al. were the first to demonstrate hemolysin production in Candida. These researchers also reported that the HLP gene was associated with hemolysin production in *C. glabrata *[36,37]. Hemolysin has a crucial role in pathogenicity as it helps pathogens acquire iron from the host’s RBC, enabling the rapid multiplication of pathogens and, thereby, the fast spread of infection. Rodrigues et al., in 2003, reported coagulase production in Candida for the first time [38]. Compared with other exoenzymes, the role of coagulase in the overall establishment and exacerbation of Candida infection still remains unclear. Therefore, the need for more studies is warranted. Research on coagulase production in Candida isolates, especially from disseminated infections, may provide future insights for understanding the pathogenesis.

Development of identification system: conventional and automation

Until recently, the mycological workup in each laboratory for the identification of Candida spp. began with the "Germ tube test." This test was first described by Reynolds and Braude in 1960 and is hence also known as the "Reynolds-Braude Phenomenon." *C. albicans* and *C. dubliniensis* share the ability to form a germ tube [39], so it cannot be used to differentiate between these two species. The germ tube lacks specificity and sensitivity​​​​​​.

Wickerham and Burton (1948) introduced the first classification system for Candida spp. based on their carbohydrate assimilation patterns. The original method was eventually replaced by the auxanographic method, which involves inoculating a minimal agar medium that lacks carbon sources with a yeast suspension. Carbohydrate solutions are placed in wells or on sterile discs on the agar surface. The yeast's ability to assimilate the carbohydrate is indicated by growth around the carbohydrate source, while the absence of growth suggests nonassimilation. In 1977, Mickelsen et al. refined the technique by modifying the inoculum density and incubation conditions, which improved the differentiation of Candida spp. Additionally, the analytical profile index, the first commercially available kit based on carbohydrate fermentation and assimilation tests, became a key tool for identifying Candida spp. [40].

The need for rapid, reliable, and easy identification methods is always warranted in diagnostic microbiological services to provide timely feedback to the treating clinician, especially when the patient is critically ill and requires urgent therapeutic intervention. In recent years, automated and molecular techniques, such as polymerase chain reaction (PCR) and matrix-assisted laser desorption ionization time-of-flight mass spectrometry, have shared a significant portion of the diagnostic portfolio. Both offer rapid and accurate pathogen identification.

Automated systems, such as the VITEK 2 Identification Yeast System (BioMérieux, Marcy-l'Étoile, France), integrate biochemical assays with database algorithms for quick and precise identification, along with AFST, providing comprehensive diagnostic support. Other AFST methods include automated systems like Sensititre YeastOne (Thermo Fisher Scientific, Waltham, MA) and Microscan (Beckman Coulter, Brea, CA), which provide rapid and standardized susceptibility profiles. Additionally, E-test strips are widely used to determine MICs through a gradient diffusion method. Colorimetric methods, such as the Alamar blue assay and flow cytometry-based assays, are emerging techniques that offer quick turnaround times for AFST. Molecular methods targeting resistance genes, such as PCR-based detection of FKS and ERG11 mutations, also contribute to rapid resistance profiling.

Emerging diagnostic tools like fluorescent in situ hybridization utilize fluorescent-labeled probes to directly visualize Candida in clinical samples, eliminating the need for culture. Nucleic acid sequence-based amplification targets RNA, offering rapid and sensitive pathogen detection. Furthermore, genotypic methods such as multilocus sequence typing, microsatellite length polymorphism, and DNA microarrays enable detailed genetic characterization of Candida spp., contributing to epidemiological studies and outbreak investigations. Serological biomarkers, including (1,3)-β-D-glucan and galactomannan, are also employed to support the diagnosis of invasive candidiasis, enhancing the overall diagnostic accuracy in clinical microbiology [40].

Renaming the Candida spp.

Recently, the nomenclature of many Candida spp. has been changed. The previous and recent names of these Candida spp. are shown in Table 2. This novel nomenclature is reflected based on recent information regarding molecular-based technologies in taxonomy, diagnostics, and epidemiology. However, these taxonomic changes have not altered the clinical relevance of these species. They continue to be associated with a wide range of infections, from superficial mucocutaneous candidiasis to severe disseminated diseases. Additionally, the growing incidence of antifungal resistance among these species presents significant challenges in clinical practice, complicating both diagnosis and treatment approaches [41].

**Table 2:** Nomenclature changes in clinically Candida spp.

Serial no.	Previous name	Recent name
1	Candida bracarensis	Nakaseomyces bracarensis
2	Candida catenulata	Diutina catenulata
3	Candida fabianii	Cyberlindnera fabianii
4	Candida colliculosa	Torulaspora delbrueckii
5	Candida famata	Debaryomyces hansenii
6	Candida glabrata	Nakaseomyces glabrata
7	Candida guilliermondii	Meyerozyma guilliermondii
8	Candida krusei	Pichia kudriavzevii
9	Candida kefyr, Candida pseudotropicalis	Kluyveromyces marxianus
10	Candida lipolytica	Yarrowia lipolytica
11	Candida lusitaniae	Clavispora lusitaniae
12	Candida nivariensis	Nakaseomyces nivariensis
13	Candida neorugosa	Diutina neorugosa
14	Candida norvegensis	Pichia norvegensis
15	Candida pararugosa	Diutina pararugosa
16	Candida pelliculosa, Pichia anomala	Wickerhamomyces anomalus
17	Candida pseudorugosa	Diutina pseudorugosa
18	Candida rugosa	Diutina rugosa

## Conclusions

The historical perspective of Candida is dynamic. Though many aspects of Candida have become clear over the years, more still need to be further elucidated to help understand the challenging mechanisms of pathogenicity, epidemiology, and antifungal resistance. Additionally, this will provide new insights for developing or designing new antifungal molecules that will enrich the antifungal armamentarium against Candida, which is currently limited to only three classes: azoles, polyenes, and echinocandins. A few new molecules, such as fosmanogepix (APX001) and arylamidine, are in phase III clinical trials and can be considered future hopes for treating drug-resistant Candida infections, especially *C. auris.*
